# Reliable mass production of *Ganaspis kimorum* (Hymenoptera: Figitidae), a larval parasitoid of *Drosophila suzukii* (Diptera: Drosophilidae)

**DOI:** 10.1093/jisesa/ieaf024

**Published:** 2025-03-14

**Authors:** Juan Huang, Rufus Isaacs, Julianna K Wilson

**Affiliations:** Department of Entomology, Michigan State University, East Lansing, MI, USA; Department of Entomology, Michigan State University, East Lansing, MI, USA; Department of Entomology, Michigan State University, East Lansing, MI, USA

**Keywords:** mass rearing, parasitoid, host availability, biological control, humidity, temperature

## Abstract

Spotted wing drosophila, *Drosophila suzukii* (Matsumura), native to Asia, has become a significant threat to soft fruit crops globally. To develop a classical biological control program for this pest, the obligate parasitoid *Ganaspis kimorum* (Buffington) was approved in the United States for field release in 2021 as a biological control agent. However, challenges in mass production and maintenance of parasitoid colonies have been common. Here, we share improved methods and offer insights into mitigating issues that limit parasitoid production. Additionally, we present a modified rearing protocol using 2-l plastic containers to produce an average of 307 *G. kimorum* per container. This information is crucial for implementing successful classical biological control programs against spotted wing drosophila with this parasitoid.

## Introduction

Spotted wing drosophila, *Drosophila suzukii* (Matsumura) (Diptera: Drosophilidae), is now a global insect pest of soft, thin-skinned fruit crops. In the first years that it became established, economic losses from *D. suzukii* for berry and cherry industries in the United States were estimated to be $500 million at an average 20% infestation rate (Bolda et al. 2010). Currently, fruit growers rely on multiple insecticide sprays during the growing season to protect their crops from this pest (Van Timmeren and Isaacs 2013, Tait et al. 2021). However, the use of insecticides can have negative impacts on the environment, nontarget organisms, and human health.

Biological control, employing natural enemies such as parasitoids, is a promising facet of integrated pest management strategies against invasive insect pests like *D. suzukii*. For example, establishment of the Asian-origin melon fly, *Bactrocera cucurbitae* (Diptera: Tephritidae) in Hawaii in 1895 resulted in introduction of the exotic larval parasitoid, *Psyttalia fletcheri* (Hymenoptera: Braconidae) (Vargas et al. 2012 and references herein). Similarly, invasion of the Oriental fruit fly, *Bactrocera dorsalis* (Diptera: Tephritidae) into Hawaii in 1945 prompted the introduction of a successful classical biological control agent, *Fopius arisanus* (Hymenoptera: Braconidae) (Clausen et al. 1965). Since then, this egg parasitoid has remained the dominant parasitoid species in Hawaii, resulting in substantial reductions of fruit fly populations (Vargas et al. 1993). Parasitoids can attack their hosts where insecticides are unable to reach and can move between different habitats. Their mobility allows them to suppress pest populations in crops as well as in adjacent non-crop habitats where the overall pest reservoir can be reduced, thereby managing pest populations more effectively.

In the pursuit of effective classical biological control agents of *D. suzukii*, a group of researchers ventured to its native habitat in Asia and discovered several larval parasitoids in several species in the genus *Ganaspis* (Hymenoptera: Figitidae) (Daane et al. 2016, Giorgini et al. 2019, Sosa-Calvo et al. 2024). These diminutive parasitoids (1.5 to 1.75 mm in length) attack early larval instars of *D. suzukii* growing inside fresh fruit, and have potential to reduce its populations (Buffington and Forshage 2016, Girod et al. 2018, Wang et al. 2018).

After rigorous risk and quality assessments under quarantine for many years (https://www.aphis.usda.gov/sites/default/files/ganaspis-brasiliensis-final-ea.pdf), *Ganaspis kimorum* Buffington (formerly *G. brasiliensis* G1 strain) received approval for field release in the United States in 2021. However, the successful implementation of a classical biological control program centered on this species hinges on the ability to mass-produce the parasitoid. Despite recent publications on laboratory colony rearing (Rossi-Stacconi et al. 2022), various research groups in the United States encountered challenges in sustaining *G. kimorum* colonies, leading to fluctuations in colony size and occasional collapses. Here, we address common issues faced during critical stages of mass rearing and outline effective remedies that have allowed for continuous rearing of this species. The issues encountered included variability in fruit quality, host availability, contaminants (ie mold and mites), and environmental conditions during incubation. A modified rearing method is described to optimize parasitoid production while minimizing associated costs. These insights are crucial for advancing the development and implementation of a robust biological control program against *D. suzukii* that includes a highly specialized parasitoid likely to require annual augmentative releases.

## Common Issues during Mass-rearing of *G. kimorum*

### Variability in Fruit Quality

Females of the *G. kimorum* only lay their eggs in *D. suzukii* larvae feeding inside fresh fruit (Seehausen et al. 2020, Sosa-Calvo et al. 2024). As such, rearing using blueberries has become the standard because they are available for purchase year-round. However, different batches of blueberries purchased at different times of the year varied in the number of eggs that *D. suzukii* flies were able to lay in the fruit. Blueberries available for purchase from local grocers between March 2022 and February 2024 originated in Michigan, Ohio, Oregon, and Florida or were imported from other countries including Peru, Mexico, Chile, and Canada and consisted of a variety of different cultivars not identified on the label. This creates inconsistency in terms of fruit firmness and underlying production practices which may contribute to variations in host availability for parasitoid oviposition. On some occasions, we suspected that insecticide residues remaining in or on blueberries, even after rinsing, were responsible for high *D. suzukii* mortality, resulting in few larvae available for parasitism. On other occasions, blueberries were not fully ripened or had tough or thicker skins, resulting in *D. suzukii* females either laying their eggs outside the fruit or ceasing egg laying, presumably because their ovipositors were unable to cut through the skin. While these challenges related to blueberries are hard to avoid, we suggest sourcing blueberries from a variety of different retailers to avoid colony collapse. Additionally, storing fruit at room temperature for a couple of days will allow underripe fruit to soften before they are offered to *D. suzukii*.

### Host Availability

The main constraint on *G. kimorum* production is host availability. *G. kimorum* females prefer first and second instar larvae of *D. suzukii* to lay their eggs (Wang et al. 2018). Ideally, *G. kimorum* parasitoids should be given blueberries infested with *D. suzukii* larvae at these stages daily, but this requires intensive labor. Thus, we developed a 5 d parasitoid ovipositional period (rearing protocol described below) that maximizes host availability. This includes (i) ensuring that *D. suzukii* adults being used are at the optimum age for maximum egg-laying capacity, and (ii) adding plenty of these egg-laying flies to the containers for initial oviposition, and (iii) allowing some of these egg-laying flies to remain in the rearing container after the parasitoids are added.

The age of *D. suzukii* adults can impact their reproductive capacity; young females lay fewer eggs compared to older ones with maximum ovipositional rates observed when females are 20 d old (Tochen et al. 2014). Furthermore, we observed that only 34% of the eggs laid by *D. suzukii* into blueberries under laboratory conditions, without any interference from parasitoids, survived to emerge as adults. Therefore, we concluded that *D. suzukii* adults used for blueberry infestation should be between 15 and 25 d old to maximize their egg output, and that we needed to increase the number of flies used for fruit infestation. We found that for each pint of blueberries, between 350 and 500 flies was optimum for maximum infestation. An indication of sufficient host population was confirmed by examining subsets of fruit for the presence of at least 5 *D. suzukii* eggs per fruit. It is also crucial to ensure that no other contaminated fruit flies such as *Drosophila melanogaster* are present during blueberry infestation since *D. melanogaster* can significantly reduce *D. suzukii* oviposition (Shaw et al. 2018).

### Mold and Mites

While *D. suzukii* is known to rely on yeast symbionts such as *Hanseniaspora uvarum* for food and development (Hamby et al. 2012), fungal contaminants can negatively affect *Drosophila* development and survival (Stensmyr et al. 2012, Chakraborty et al. 2022). Mold readily grows on blueberries, especially as they continue to decay during the 3 to 4 wk incubation period necessary to allow *G. kimorum* to complete its lifecycle. Throughout our rearing of *G. kimorum*, we frequently encounter two types of molds affecting parasitoid survival: white cotton-like mold (possibly *Botrytis*) and greenish powdery mold (possibly *Aspergillus*). The mycelial growth of the white mold can be detrimental to parasitoids by trapping them as they emerge or entangling their tarsi upon contact, whereas the spores of the green powdery mold stick to their bodies, preventing them from walking and flying freely. Previous protocols recommended rinsing blueberries in a bleach solution before rinsing with water to reduce mold growth (Rossi-Stacconi et al. 2022); however, we found that bleached blueberries were more prone to mold growth than those without. Chakraborty et al. (2022) demonstrated that inoculation of raspberries with *H. uvarum* entirely suppressed the development of the gray mold, *Botrytis cinerea* which had negative impacts on *D. suzukii* larval development and oviposition. However, baker’s yeast, *Saccharomyces cerevisiae* is more readily available, and we found this also inhibited mold growth when sprinkled onto the surface of blueberries before infestation, as was recommended in an earlier protocol (Rossi-Stacconi et al. 2022). In addition, we found that covering the blueberries with moist paper towel after parasitoid oviposition helped reduce the spread of mold within and among rearing containers as they were being handled. We also observed less mold growth in rearing containers that were heavily infested with *D. suzukii* larvae, which might be due to a combination of factors including the density of larvae feeding in the fruit (Wertheim et al. 2002) or the acidity of the juices released during berry degradation (Smars et al. 2002) since organic acids have antimicrobial properties (Sundberge and Jönsson 2005).

Mites can be another challenge to the overall health and success of a parasitoid colony. Mite infestations can be hard to detect and are easily missed. Additionally, once mites are established, eradication is virtually impossible. Therefore, we isolate any field exposed containers or collections from our colony containers and regularly inspect adult parasitoids collected into holding vials. We also take great care in washing and sterilizing rearing containers using soap and bleach between cohorts.

### Rearing Conditions

Temperature and humidity affect the development, survival, and oviposition of insects, and maintaining optimum environmental rearing conditions is critical for *G. kimorum* development and survival. When parasitized *D. suzukii* larvae feed and develop inside blueberries, excess liquid is produced from the breakdown of the infested berries. To prevent excess moisture in rearing containers, we found it useful to layer paper towels at the bottom of rearing containers. This absorbs excess liquid and helps prevent drowning of adult parasitoids and fly larvae. In addition, the paper towels become pupation substrates and help retain moisture over time during the incubation period. However, condensation caused by temperature and humidity fluctuations can still lead to sufficient moisture on the container surfaces to cause parasitoids to drown.

Hougardy et al. (2019) reported that *Ganaspis brasiliensis* Ihering completes development when temperatures are between 17.2 and 27.5 °C, and that development time decreases with increasing temperature, though females accept a wide range of temperatures of 19.9 to 30.6 °C for oviposition. While these parasitoids have a range of acceptable temperature conditions, the optimum temperature for rearing should consider other factors such as mold growth and fruit deterioration. High temperatures accelerate host larval growth to stages unsuitable for parasitoid oviposition and also promote mold growth in this humid environment. Thus, we recommend temperatures between 20 and 23 °C to encourage parasitoid oviposition and development while also inhibiting the fungal decay of blueberries.

Relative humidity is another key factor significantly impacting parasitoid survival, especially when parasitized host larvae wander in search of suitable pupation sites. *D. suzukii* larvae and pupae are sensitive to desiccation (personal observation). Since parasitoids are developing inside the host puparium, it is critical to keep the paper towel moist in rearing containers and to keep the rearing room between 60% and 65% relative humidity to prevent desiccation. One study found that the survival rate of *Leptopilina japonica* (Hymenoptera: Figitidae), another larval parasitoid of *D. suzukii* now found from Michigan to Northeastern US (Gariepy et al. 2024), reduced with decreasing relative humidity, and all parasitized hosts died once relative humidity was at 33% (Murata et al. 2013). However, we observed that relative humidity over 70% also led to mold overgrowth and condensation in rearing containers. Therefore, balancing relative humidity levels alongside temperature considerations is crucial for maintaining optimal rearing conditions for *G. kimorum*. Thus, we recommend maintaining relative humidity between 60 to 65% as an optimum condition for *G. kimorum* rearing.

## Protocol for Mass Rearing *G. kimorum* using Large Containers

**Fig. 1.**
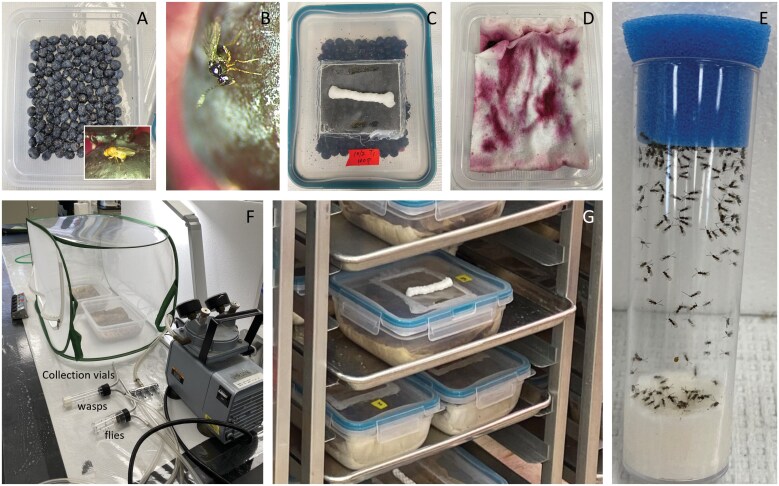
Illustration of key steps and equipment used in mass rearing *Ganaspis kimorum*. Single layer of blueberries with yeast for fly infestation (A); *G. kimorum* ovipositing into infested blueberry (B); rearing lid with honey and saturated dental wick (C); paper towel layer on top of infested blueberries during incubation (D); parasitoid holding vial with densely packed roll of saturated paper towel and blue plug with honey (E); mesh cage for releasing parasitoids and flies from rearing containers with a vacuum pump aspirator and splitter connecting two collection vials (F); and rearing containers on a rolling cart during incubation (G).

**Fig. 3.**
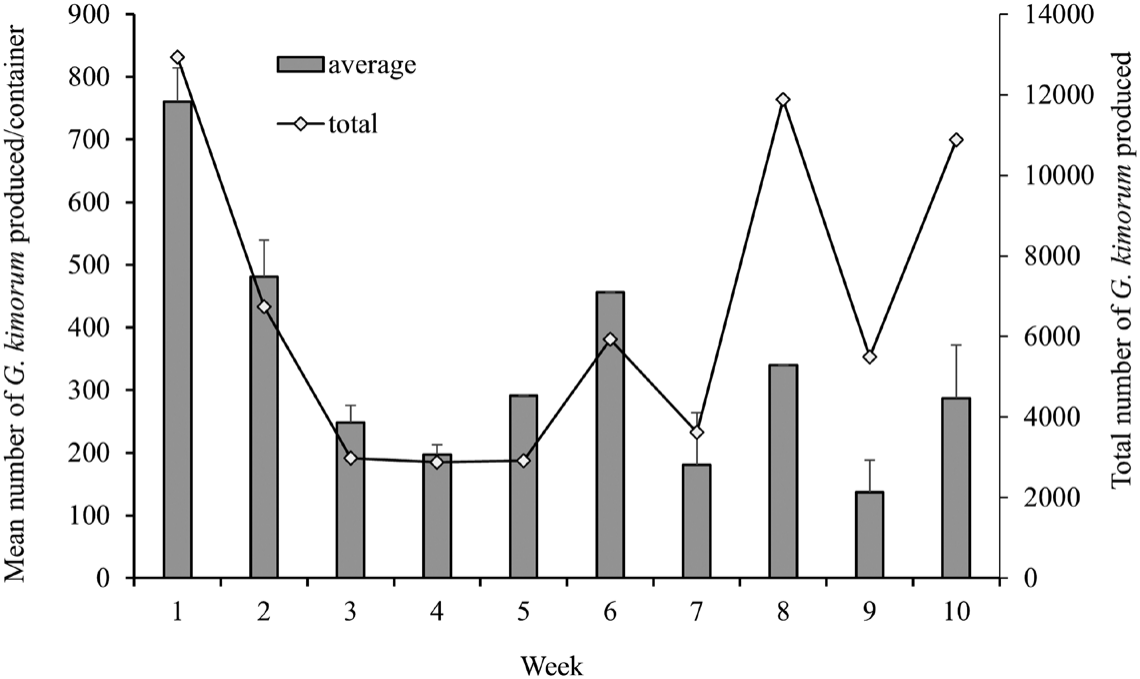
Mean + SEM numbers of *Ganaspis kimorum* produced per rearing container and total numbers of parasitoids produced weekly using modified rearing protocol.

Here we describe in detail the protocol we have used with great success for mass-rearing *G. kimorum.* This approach has allowed for the production of 71,436 parasitoids with 477 h of labor over a period of 3 mo. We use 2-l rearing containers (21 L × 16.5 W × 7 H cm^3^) (Snapware, Instant Brands Inc., Greencastle, PA) with a rectangular hole (8.5 × 10 cm^2^) cut into the lid to accommodate a piece of fine mesh for ventilation (Fig. 1). Following the protocol below for infestation and egg laying, containers of this size accommodate about 1 pint of blueberries in a single layer and these produce an average of 307 *G. kimorum* per container each week (Fig. 3).

Infesting blueberries with *D. suzukii*

1. Inspect blueberries and discard any that appear damaged or overly soft, rinse blueberries with cold tap water and soak them for 30 min; then repeat this process for 2 more times.
2. Line each container with 4 layers of Viva (Kimberly-Clark, Neenah, WI) paper towel (18 × 14.5 cm^2^, signature cloth paper towel). In our experience, this is the brand that performs best for absorbing and retaining liquids. Ensure the paper towel is saturated with water but not dripping by adding 45 ml dH_2_O evenly and pressing the towel against the bottom and sides of the container.
3. Add a single layer of blueberries (approx. 1 pint) on top of the paper towel.
4. Sprinkle 2 to 3 pinches of Fleischmann’s instant yeast evenly onto blueberries, ensuring each fruit is in contact with yeast; if blueberries are dry, spray distilled H_2_O on fruit prior to yeast application to help the yeast stick.
5. Let fruit air dry for ~10 min before adding 300 to 350 mixed sex *D. suzukii* in each container for oviposition.

Parasitoid oviposition

1. After 2 d, remove about 70% to 80% of the *D. suzukii* flies from each container, leaving some (<30%) behind to provide a continuous supply of host larvae of the right age for parasitoids during their 5 d egg laying period.
2. Depending on parasitoid availability, add 50 to 100 pairs of male and female parasitoids to each container. Seal the mesh lid, place a moist cotton wick to provide a water source on top, and with a small paint brush, add 2 streaks of honey to provide a food source for the egg laying parasitoids. Parasitoids are allowed to oviposit for 5 d.
3. After 5 d, prepare 50 ml collection vials (see protocol below). In a fine mesh cage (34 × 34 × 61 cm, Mantid Kingdom, Beaverton, OR), open each container to release the flies and parasitoids. Use an aspirator attached to a vacuum pump to remove any remaining flies and parasitoids from each container and the parasitoids from the walls of the mesh cage, and collect these parasitoids into the prepared 50 ml vials. These parasitoids can be either stored in a cold chamber (16 °C) for a couple of weeks until needed for experiments or used again for the next 2 to 3 rounds of parasitoid oviposition. The flies are discarded.
4. After all adult flies and parasitoids are removed, cover the layer of blueberries with 2 layers of Viva paper towels. One end of this towel layer should be tucked underneath the bottom layer of paper towel to wick up excess juice from the bottom; mold growth will be inhibited when blueberries are entirely covered. Check the containers twice a week and if they are becoming dry, spray the paper towels with a small volume of distilled water as needed over the duration of the pupation period.
5. About 1 wk later, flies will start to emerge. Release them into a cage at least every other day until parasitoids begin to emerge to avoid flies re-infesting the blueberries. In the meantime, inspect each container to see if the top layer paper towel needs additional water (see above). Containers that are too dry will decrease parasitoid survival.
6. About 3 wk after the initial date of parasitoid oviposition depending on the rearing temperature, male parasitoids should begin to emerge. At this point, add 2 additional streaks of honey on the mesh portion of lids to provide food for the emerging parasitoids.
7. Newly emerged parasitoids should be collected from the containers into prepared collection vials with a honey source as described below, at least twice per week.
8. About 2 wk after initial parasitoid emergence, or when no more parasitoids have emerged for a week, freeze the sealed containers for at least 24 h before cleaning them in preparation for the next round. In a separate room, soak the container bottoms and lids in 7% v/v bleach water and dish soap for 2 h, then rinse them thoroughly with warm water before drying them.

Preparing vials for collecting and holding parasitoids

1. Parasitoid holding vials are prepared using 50 ml polystyrene Drosophila vials (Genesee Scientific, CA), a blue foam plug, and a tightly rolled strip of Viva paper towel pressed down firmly into the bottom of each vial (1.5 cm high) (Fig. 1). When ready to use, raw honey (50 to 100 µl) is dabbed with a small paint brush onto the center of the plug and the paper towel is saturated with dH_2_O.
2. To saturate the paper towel, add dH_2_O to each vial until there is standing water on top. Let the water soak in for at least 10 min. Afterwards, dump any excess water, and pack the paper towel tightly using a gloved finger. Remove excess water on all surfaces inside the vial with dry paper towels. Make sure there are no water droplets on the wall, otherwise parasitoids can drown.
3. Each vial can accommodate 50 female and 30 to 50 male parasitoids. Collect parasitoids into prepared vials with a Gast compressor/vacuum pump (10 to 15 psi).
4. If collected parasitoids will not be used for egg laying immediately, they can be stored in a 16 °C growth chamber for at least 14 d, with weekly vial changes. However, before cold storage they must be allowed to feed on raw honey for 2 d at room temperature. Immediately placing them in 16 °C prevents them from feeding appropriately before storage. Cold stored parasitoids should be switched to newly prepared holding vials with fresh honey weekly. Parasitoids need to be taken out of the cold chamber 1 d prior to egg laying so they are ready to lay eggs.

## Production Refinement Experiments

Here we describe a series of experiments conducted to fine tune the protocols related to oviposition duration, female age, and the effects of cold storage on overall *G. kimorum* fecundity. Preparation and handling of rearing containers followed the protocol described above. All experiments were conducted in a rearing room maintained at 21 °C and 60% relative humidity with a 16:8 (L:D) light cycle during oviposition.

### Oviposition Duration

To determine how oviposition duration affected parasitoid production, 100 mated females of ca. 7 to 8 d old *G. kimorum* were transferred to rearing containers with *D. suzukii*-infested blueberries and were allowed to oviposit for either 2 or 5 d periods. Thirteen rearing containers were randomly assigned to the 2 d period; another 12 containers were assigned to the 5 d period. Progeny produced per treatment was compared, as described below.

### Effect of Parasitoid Age on Production

To determine how parasitoid age affected fecundity, parasitoids collected from different days were compared. Thirty rearing containers with *D. suzukii*-infested blueberries were divided into 3 groups (10 containers/group). Each container within the same group received 50 pairs of male and female parasitoids that were either newly collected (1 to 2 d old), 5 d old, or 10 d old. Progeny produced per treatment were collected and compared as described below.

### Effect of Cold Storage on Production

To determine how cold storage affected future fecundity, parasitoids collected from multiple days were held at 21 °C for 2 d to allow them to feed on honey and mate, and then transferred into a growth chamber maintained at 16 °C and 60% relative humidity with a 16:8 L:D light cycle for 10 to 14 d. Twenty rearing containers with *D. suzukii*-infested blueberries were divided evenly into 2 groups, one group receiving 50 pairs of cold stored parasitoids per container, the other receiving 50 pairs of 5 to 6 d old parasitoids held at 21 °C since their collection. Progeny from each treatment were recorded and compared, as described below.

### Data Analysis

All data were tested and met assumptions of homoscedasticity and normality (Shapiro–Wilk test), hence treatments were compared by analysis of variance (SAS Institute 2020). Mean separations were performed via Tukey’s HSD (*α* = 0.05).

## Results of the Production Refinement Experiments

**Fig. 2.**
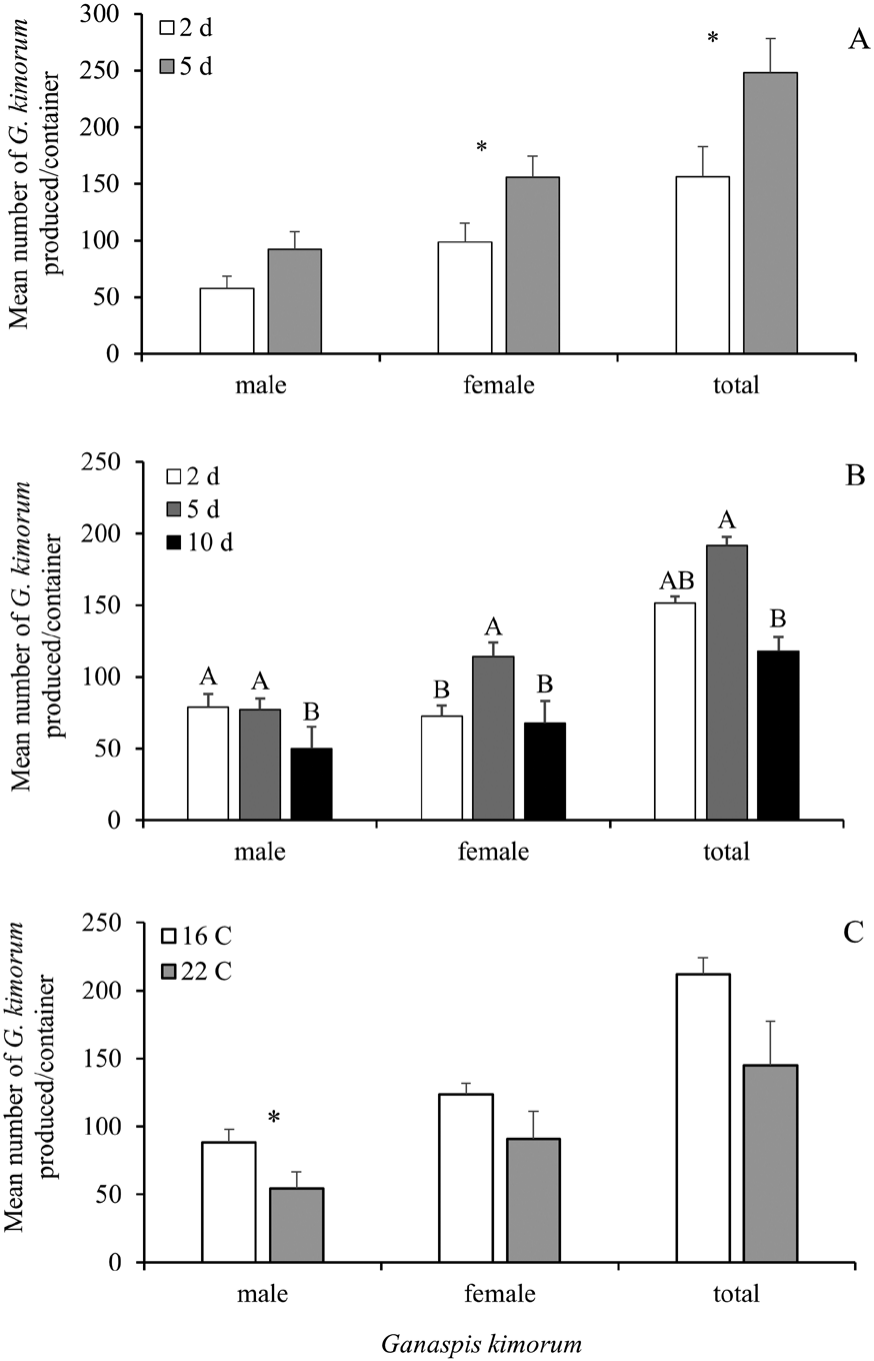
Mean + SEM numbers of male, female, and total *Ganaspis kimorum* produced per rearing container by 100 females laying eggs for 2 and 5 d (A), 50 females at 2, 5, or 10 d old (B), and 50 females stored at 16 or 22 °C (C). Asterisk indicates a significant difference between treatments (*P* ≤ 0.05). Means topped with different letters differ significantly at *P* = 0.05.

When parasitoids were allowed to lay eggs for 5 d as opposed to 2 d, they produced significantly more females (*F* = 5.41, df = 1, 23, *P* = 0.03) and total number of parasitoids (*F* = 5.41, df = 1, 23, *P* = 0.03) and numerically more males (*F* = 3.53, df = 1, 23, *P* = 0.07) (Fig. 2A). The age of egg-laying parasitoids did impact the number of progeny produced (*F* = 4.89, df = 2, 27, *P* = 0.02); 2 and 5 d old parasitoids produced a similar number of males, more than the 10 d; however 5 d old parasitoids produced significantly more females than the other aged treatments (Fig. 2B). Ten d old parasitoids produced the lowest number of both males and females, significantly less than 5 d old females. The highest parasitoid production was by 5 d old females, followed by the 2 d and the 10 d old. Parasitoids held in cold storage at 16 °C produced significantly more males (*F* = 4.83, df = 1, 18, *P* = 0.04) but a similar number of females (*F* = 3.13, df = 1, 18, *P* = 0.09) compared to those subjected to 22 °C (Fig. 2C). These results supported improved mass rearing methods that included stretching the interval between container transfers, targeting the optimum age for maximizing egg-laying, and banking parasitoids during weeks when production levels were robust.

## Overall Production Levels Using the Modified Rearing Protocol

Mass releases of parasitoids require production of large numbers to maintain the colony while also producing excess for release. In 2023, we aimed to produce 39,000 for release as part of a USDA-funded project to evaluate *G. kimorum* for regulation of *D. suzukii* populations. Between 31 May and 1 September 2023, using the modified rearing method described above and 477 h of labor (not including *D. suzukii* rearing), we produced 32,348 males and 39,088 females for a total of 71,436 parasitoids from 238 rearing containers. This surpassed our goal of 39,000 individuals for release in 30 cherry and/or blueberry sites in Michigan in 2023 (Fig. 3). On average, each container produced 307 ± 1.2 parasitoids (mean ± SEM) (134 ± 0.6 males and 154 ± 0.6 females) with a median at 236. The reduction in parasitoid production per container after the first 1 to 2 wk was mainly due to low host availability caused by using less than 14 d old *D. suzukii* for oviposition and mold contamination. The total number of parasitoids produced weekly ranged from 2,909 to 12,937 depending on the number of rearing containers set up and the quality of the blueberries available at the time.

## Discussion

Our modified rearing protocol allowed us to readily produce thousands of *G. kimorum* weekly (Fig. 1). Furthermore, based on the target number of parasitoids desired, we can now confidently determine the number of rearing containers and other supplies needed to achieve production goals. We found that 5 to 6 d old parasitoids, provided with 5 d to oviposit, will maximize egg laying and production of female progeny. We also found that we can hold parasitoids that emerge asynchronously in cold storage for at least 14 d without a significant reduction in fecundity. Mass rearing of *G. kimorum* using fresh fruit will continue to be challenging unless an artificial diet or other alternative rearing substrates are developed. However, mass production of this parasitoid in blueberries is achievable with regular monitoring and quality control measures that allow for timely interventions to minimize the impact of fruit quality, host availability, and colony contaminants.
